# Supplementary material for: The Papain-like Protease Domain of Severe Acute Respiratory Syndrome Coronavirus 2 Conjugated with Human Beta-Defensin 2 and Co1 Induces Mucosal and Systemic Immune Responses against the Virus
Source: Vaccines (Basel). 2024 Apr 19;12(4):441. doi: 10.3390/vaccines12040441 (PMC11053661; doi:10.3390/vaccines12040441)
Supplement: Supplementary file 1 [file vaccines-12-00441-s001.zip › vaccines-2923529-supplementary.pdf]

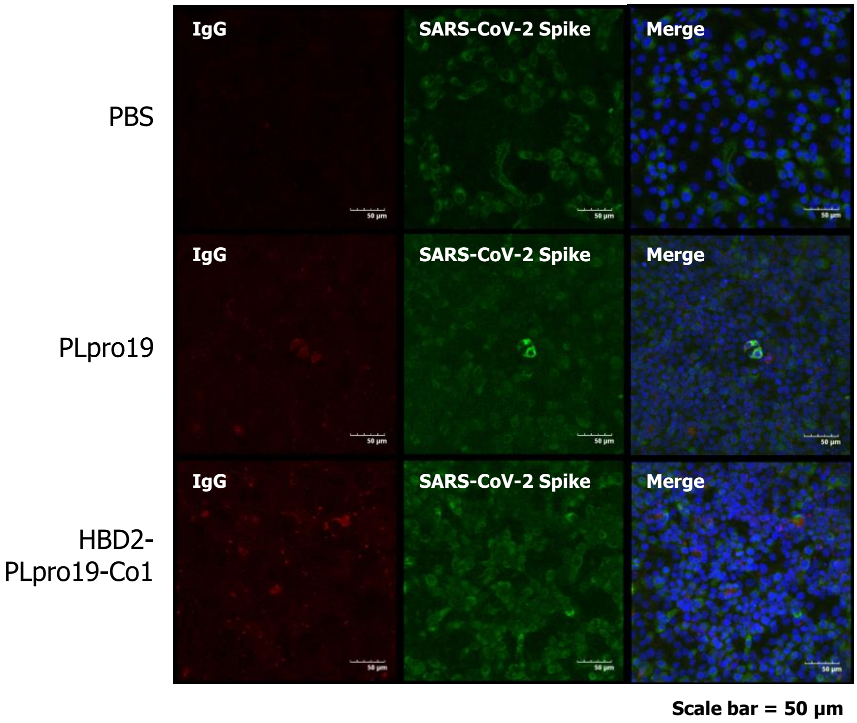

Figure S1. Interaction between infected Vero E6 and the sera of mice immunized with recombinant PLpro19 proteins. PLpro19-specific IgG from the sera of mice immunized with PLpro19 or HBD2-PLpro19-Co1 was incubated with Vero E6 cells for 1 h after their infection with SARS-CoV-2 (Wuhan strain). Mouse IgG (red) and SRAS-CoV-2 spike (green) were detected by confocal laser scanning microscopy. Scale bars = 50 μm.

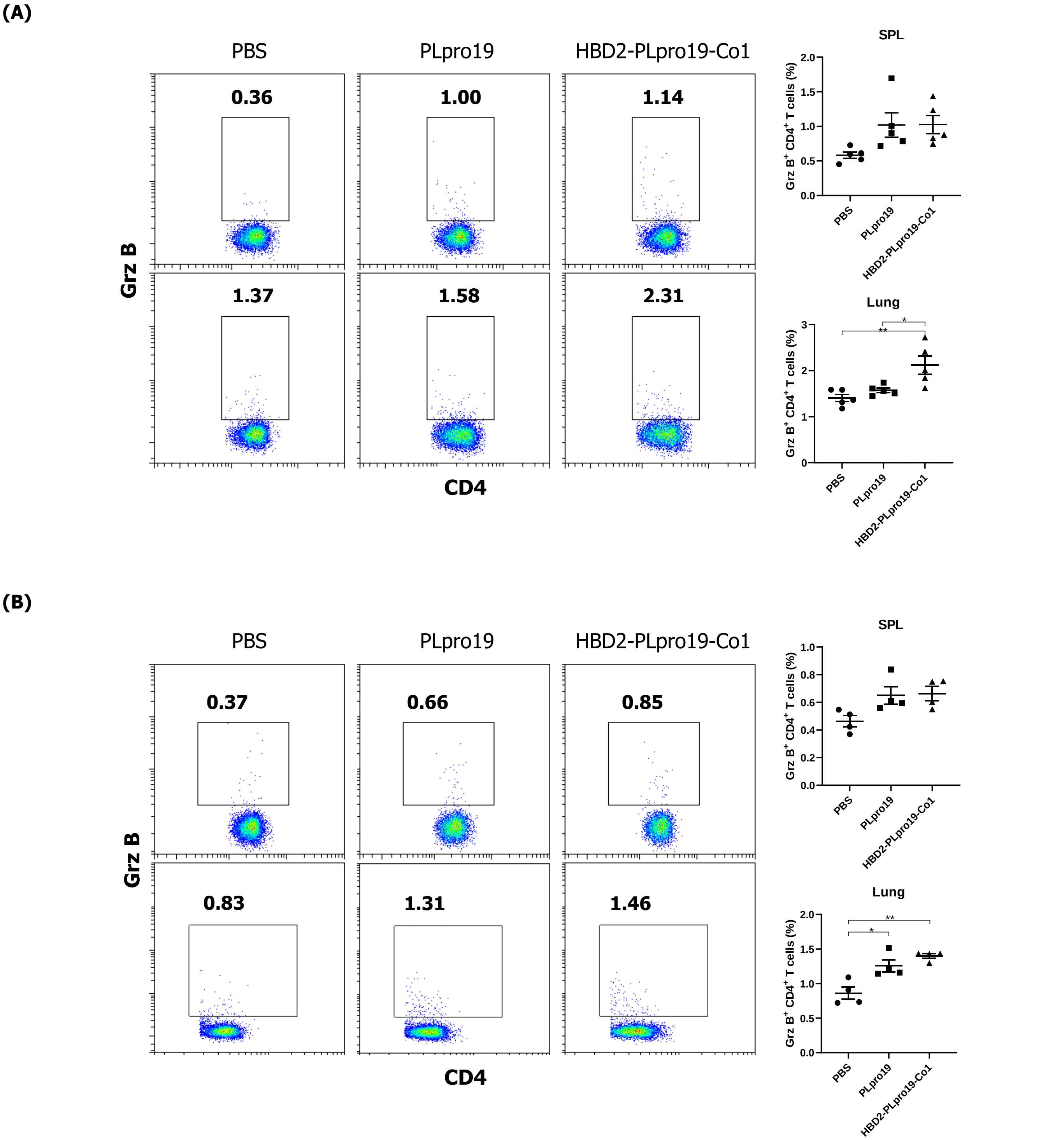

Figure S2. Cytotoxic CD4$^+$ T-cells in hACE2 KI mice. (A) hACE2 KI mice were immunized intranasally with recombinant PLpro19 proteins five times. One week after the last immunization, cells were isolated from the spleen and lung and stimulated with PLpro19 protein (1 μg) for 24 h. Grz B$^+$ CD4$^+$ T-cells were detected by flow cytometry. (B) Two months after the last immunization, hACE2 KI mice were boosted with 10 μg of PLpro19 protein. One week after boosting, cells were isolated from the spleen and lung and stimulated with PLpro19 protein (1 μg) for 24 h. Grz B$^+$ CD4$^+$ T-cells were detected by flow cytometry. All data were analyzed in a one-way ANOVA (*$p < 0.05$, **$p < 0.01$). The data are presented as the mean ± SE.
